# Levodopa Versus Dopamine Agonist after Subthalamic Stimulation in Parkinson's Disease

**DOI:** 10.1002/mds.28382

**Published:** 2020-11-09

**Authors:** Marina Picillo, Onanong Phokaewvarangkul, Yu‐Yan Poon, Cameron C. McIntyre, Sinem Balta Beylergil, Renato P. Munhoz, Suneil K. Kalia, Mojgan Hodaie, Andres M. Lozano, Alfonso Fasano

**Affiliations:** ^1^ Department of Medicine, Surgery and Dentistry, Neuroscience Section, Centre for Neurodegenerative Diseases (CEMAND) University of Salerno Salerno Italy; ^2^ Department of Medicine, Faculty of Medicine, Chulalongkorn Centre of Excellence for Parkinson's Disease & Related Disorders Chulalongkorn University and King Chulalongkorn Memorial Hospital, Thai Red Cross Society Bangkok Thailand; ^3^ Edmond J. Safra Program in Parkinson's Disease, Morton and Gloria Shulman Movement Disorders Clinic Toronto Western Hospital Toronto Ontario Canada; ^4^ Department of Biomedical Engineering Case Western Reserve University Cleveland Ohio USA; ^5^ Division of Neurology University of Toronto Toronto Ontario Canada; ^6^ Krembil Research Institute Toronto Ontario Canada; ^7^ Division of Neurosurgery University of Toronto Toronto Ontario Canada; ^8^ Center for Advancing Neurotechnological Innovation to Application (CRANIA), Toronto Ontario Canada

**Keywords:** Parkinson's disease; deep brain stimulation; antiparkinsonian medications; levodopa; dopamine agonist

## Abstract

**Background:**

No clinical trials have been specifically designed to compare medical treatments after surgery in Parkinson's disease (PD).

**Objective:**

Study's objective was to compare the efficacy and safety of levodopa versus dopamine agonist monotherapy after deep brain stimulation (DBS) in PD.

**Methods:**

Thirty‐five surgical candidates were randomly assigned to receive postoperative monotherapy with either levodopa or dopamine agonist in a randomized, single‐blind study. All patients were reevaluated in short‐ (3 months), mid‐ (6 months), and long‐term (2.5 years) follow‐up after surgery. The primary outcome measure was the change in the Non‐Motor Symptoms Scale (NMSS) 3 months after surgery. Secondary outcome measures were the percentage of patients maintaining monotherapy, change in motor symptoms, and specific non‐motor symptoms (NMS). Analysis was performed primarily in the intention‐to‐treat population.

**Results:**

Randomization did not significantly affect the primary outcome (difference in NMSS between treatment groups was 4.88 [95% confidence interval: −11.78–21.53, *P* = 0.566]). In short‐ and mid‐term follow‐up, monotherapy was safe and feasible in more than half of patients (60% in short‐ and 51.5% in mid‐term follow‐up), but it was more often possible for patients on levodopa. The ability to maintain dopamine agonist monotherapy was related to optimal contact location. In the long term, levodopa monotherapy was feasible only in a minority of patients (34.2%), whereas dopamine agonist monotherapy was not tolerated due to worsening of motor conditions or occurrence of impulse control disorders.

**Conclusions:**

This trial provides evidence for simplifying pharmacological treatment after functional neurosurgery for PD. The reduction in dopamine receptor agonists should be attempted while monitoring for occurrence of NMSs, such as apathy and sleep disturbances. © 2020 The Authors. *Movement Disorders* published by Wiley Periodicals LLC on behalf of International Parkinson and Movement Disorder Society

Deep brain stimulation (DBS) of the subthalamic nucleus (STN) is an established treatment for patients with Parkinson's disease (PD) complicated by motor fluctuations and dyskinesias.^1^ STN DBS improves levodopa‐responsive motor signs, thus allowing a reduction in antiparkinsonian medications, which, in turn, alleviates levodopa‐induced dyskinesias.^1^ Considerable variation in the extent of dopaminergic drug reduction has been reported in short‐ and medium‐term follow‐up, with values ranging from 20% to 100%.^2^, ^3^ Most studies have described the pharmacological therapy exclusively in terms of levodopa equivalent daily dose (LEDD), with no further specification of the different agents being used.^4^

Although simplification of the medication regimen is advised and constantly seen in the immediate postoperative period following STN DBS, no formal studies have been conducted to provide evidence‐based criteria to guide physicians in this regard. In spite of the steadily expanding use of DBS in PD patients, very few (and only retrospective) studies have been performed to explore medication adjustments following DBS.^4^ A retrospective analysis reported that more than 30% of patients were in monotherapy with either levodopa (LD) or dopamine receptor agonists (DA) at 1‐year follow‐up after STN DBS.^5^ Some of these studies have highlighted a very important consequence of inadequate medication adjustments after surgery, namely the occurrence of DA withdrawal syndrome and its role in postsurgical depression and the risk of suicidality.^6^, ^7^

To date no clinical trials have been done specifically to compare the efficacy and safety of LD versus DA monotherapy in terms of motor and non‐motor symptoms (NMS) after STN DBS. As such, the management of antiparkinsonian medications after STN DBS depends mainly on a neurologist's personal experience or patient preference, with the exception of some pragmatic recommendation on postoperative issues provided by the International Parkinson and Movement Disorders Society in 2006.^8^

The aim of the present randomized, prospective, single‐blind trial is to compare the efficacy and safety of LD monotherapy versus DA monotherapy after STN DBS over different follow‐up periods. Our hypothesis is that, as opposed to LD, DA may be more effective than LD in treating NMS at the risk of inducing impulse control disorders (ICDs) and poorer motor control.^9^

## Patients and Methods

Patients were eligible for enrollment if they had received a clinical diagnosis of idiopathic PD according to the British Parkinson's Disease Society Brain Bank criteria,^10^ had been candidates for STN DBS, were under treatment with both LD and oral DA (either pramipexole or ropinirole) at the time of surgery, and could provide informed consent. Candidacy for STN DBS is detailed elsewhere with well‐established criteria.^11^, ^12^ Briefly, patients needed to be diagnosed with PD for at least 5 years, had no contraindications for surgery, were under age 70 years, had disabling parkinsonian motor symptoms and/or dyskinesias despite optimal medical therapy, and had no dementia or major psychiatric illness. In keeping with the protocol in place at our center,^12^ patients with active ICDs were not included in the trial, as they do not qualify for DBS.

### Study Design and Outcomes

This study was a randomized, single‐blinded trial comparing LD monotherapy with DA monotherapy (either pramipexole or ropinirole) after STN DBS in PD patients. The evaluating clinician was blinded to treatment arm, whereas patients and prescribing clinicians were unblinded.

The trial was conducted at the Movement Disorders Centre of Toronto Western Hospital (TWH), Toronto, Ontario, Canada (clinicaltrials.gov identifier: NCT02347059). The protocol was approved by the local ethics committee, and all patients provided written informed consent before enrollment.

Patients were enrolled in pairs, with one patient randomly assigned to LD monotherapy and the other to DA monotherapy after the surgical procedure (1:1). Randomization, monitoring, and data management were performed locally. Randomization was performed using an online list randomizer (https://www.random.org/lists). The study coordinator generated the random allocation sequence before enrollment. Then, the prescribing clinician enrolled participants in the outpatient clinic, and the study coordinator assigned each participant to the intervention.

Given the well‐established notion that STN DBS effectively treats motor signs, the primary endpoint was the NMS outcome 3 months after surgery compared to baseline (preoperative), as assessed using the Non‐Motor Symptoms Scale total score (NMSS).^13^ In line with our hypothesis, we expected DA monotherapy would produce a greater improvement in the NMSS compared to LD monotherapy. Secondary outcome measures evaluated 3 months after surgery included the (1) percentage of patients maintaining monotherapy; (2) motor symptoms, as assessed using the Unified Parkinson's Disease Rating Scale, Part III (UPDRS‐III);^14^ (3) activities of daily living as rated using the Unified Parkinson's Disease Rating Scale, Part II (UPDRS‐II)^14^; (4) motor fluctuations and dyskinesias as assessed using the Unified Parkinson's Disease Rating Scale, Part IV (UPDRS‐IV) (14);^14^ (5) quality of life as assessed using the 39‐Item Parkinson's Disease Questionnaire (PDQ‐39) summary index;^15^ (6) severity of anxiety and depression as evaluated using the Hospital Anxiety Depression Scale—anxiety subscore and Hospital Anxiety Depression Scale—depression subscore (HAD‐A and HAD‐D), respectively;^16^ (7) severity of apathy as rated using the Apathy Evaluation Scale, both self‐administered (AESs) and caregiver‐administered (AESc);^17^ (8) severity of ICDs as rated using the Questionnaire for Impulsive‐Compulsive Disorders in Parkinson's Disease (QUIP);^18^ and (9) severity of sleep disorders, as evaluated using the Parkinson's Disease Sleep Scale (PDSS).^19^ UPDRS‐II and UPDRS‐III were collected on medication at baseline and on medication/on‐stimulation after DBS.

To compare the effects of changes in antiparkinsonian medications, LEDD was calculated according to established methods and reported as total, further divided as derived from DA or LD (LEDD DA [levodopa equivalent daily dose dopamine agonist] and LEDD LD [levodopa equivalent daily dose levodopa]), respectively.^20^ As such, a 100‐mg daily dose of standard LD was equivalent to the following doses of other medications: 75 mg of controlled‐release levodopa, 133 mg of levodopa plus entacapone, 1 mg of pramipexole, 5 mg of ropinirole, 10 mg of selegiline, and 1 mg of rasagiline.^20^

Safety was assessed by recording the frequency and severity of reported adverse events. Any new symptom or worsening of a preexisting symptom was classified as an adverse event.

### Interventions

Enrolled patients underwent STN DBS according to the standard practice used at TWH.^21^, ^22^ Quadripolar electrodes (model 3387) connected to an implantable pulse generator (Activa PC or SC, Medtronic, Dublin, Ireland) were used for all patients.

In keeping with TWH protocols,^21^, ^22^ dopaminergic treatment was unchanged for the first month after surgery. During their first programming visit, patients were randomly assigned to either LD or DA monotherapy. Patients randomly assigned to LD gradually weaned off DA, whereas those randomly assigned to DA slowly weaned off LD. The time to discontinue DA or LD varied and depended on the initial dose, generally ranging from 1 to 4 weeks. During the trial phase, further adjustments (including increases) of the allocated drug were possible. Then, every patient was seen once a week for the subsequent 8 weeks to optimize both stimulation and medical treatment. All enrolled patients were reassessed 3 months after surgery. Importantly, patients unable to maintain LD or DA monotherapy were kept at the lowest‐possible dose of the drug that they were assigned to discontinue. The ability to maintain monotherapy as well as adverse events was also verified 6 months after surgery. Finally, medications, motor status, and adverse events were openly evaluated in the long‐term, 2.5 years after study enrollment.

### Electrode Location and Stimulation

All patients underwent a postoperative brain MRI within 1 week after surgery. We generated patient‐specific anatomical models of the STN using an academic DBS research software tool.^23^, ^24^ A 3D visualization of both the implanted DBS electrodes and the volumes of the STN in the left and right hemispheres was constructed for each patient using pre‐ and postoperative T1‐weighted MRIs. The distance between the active electrode contact and the center of the STN was then calculated for the left and right electrodes along the *x*‐axis (medial–lateral), *y*‐axis (anterior–posterior), and *z*‐axis (superior–inferior).

### Statistical Analysis

Based on earlier treatment results,^25^ we determined that at least 20 patients (using a 2‐sided test with an α‐value of 0.05 and a β‐value of 0.2 and assuming a dropout rate of 20%) would need to be enrolled in each treatment group to detect a mean difference of 0.5 SD (standard deviation) in the primary outcome between the groups.

*t* Tests or χ^2^ tests were used for group comparisons as appropriate at different time points. For efficacy analysis, generalized linear models were applied with treatment as a fixed factor. Statistical analysis was primarily by intention‐to‐treat and secondarily per‐protocol.

A Kaplan–Meier survival analysis was performed to estimate the time to monotherapy failure for the 2 treatment arms, which were compared by means of the log‐rank test. Finally, we performed multiple univariate logistic regression analysis to identify predictors of 3‐month postoperative monotherapy failure (independent variables explored: age, disease duration, UPDRS‐III, LEDD, LEDD LD, LEDD DA, weight, randomization, active DBS electrode contact‐STN distance along the *x*–*y*–*z* axes averaged over the hemispheres). If any variables were found to be significant at the *P* < 0.05 level, they were assessed together in a single multivariate regression analysis with a *P* < 0.05 inclusion level.

The significance level was set at *P* ≤ 0.05. Analyses were run using SPSS version 23.0 (IBM, Chicago, IL).

## Results

### Study Population

**FIG. 1:**
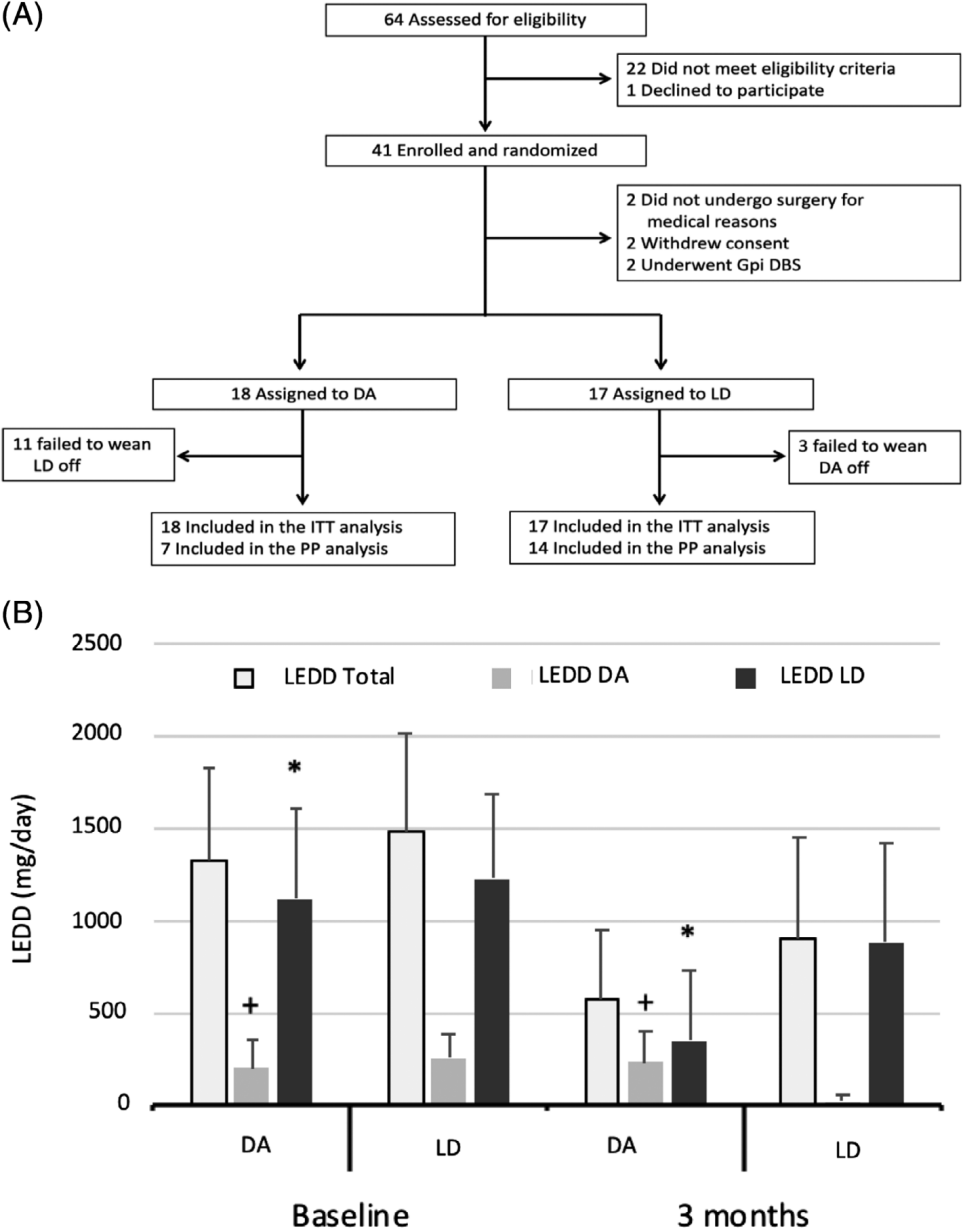
(**A**) Flow diagram and study design. Abbreviations: DA, dopamine receptor agonists; Gpi DBS, deep brain stimulation of globus pallidus pars interna; ITT, intention‐to‐treat; LD, levodopa; PP, per‐protocol. (**B**) Three‐month reduction in LEDD total, LEDD DA, and LEED LD in treatment groups (intention‐to‐treat analysis). +, greater increase in LEDD DA in DA treatment group compared to LD treatment group (*P* = 0.035); *, greater reduction in LEDD LD in DA treatment group compared to LD treatment group (*P* = 0.019); DA, dopamine receptor agonist; LD, levodopa; LEDD, levodopa equivalent daily dose; LEDD DA, levodopa equivalent daily dose dopamine agonist; LEDD LD, levodopa equivalent daily dose levodopa.

Between December 1, 2014, and December 31, 2016, 64 patients were screened, 41 patients were enrolled, and 35 were randomly assigned to LD (17) or DA (18) (Fig. 1A). All patients underwent bilateral surgery except 3 who underwent unilateral procedures (1 in the left STN and 2 in the right STN).

There were no significant differences in baseline characteristics between the 2 groups (Table 1). Also, there were no significant baseline differences in either primary or secondary outcome measures between the 2 groups (*P* > 0.05). Although DA tended to show higher NMSS compared to LD, the difference was not significant (*P* = 0.574).

**TABLE 1:** Baseline characteristics of the intention‐to‐treat cohort before surgery

	LD (N = 17)	DA (N = 18)	*P*
Age (yr)	61.47 ± 8.23	57.44 ± 9.24	0.184
Men, n (%)	12 (70.6)	14 (77.8)	0.711
Disease duration (yr)	11.94 ± 4.32	10.61 ± 3.69	0.333
NMSS	59.83 ± 46.08	43.42 ± 25.31	0.574
UPDRS‐III (med on)	12.16 ± 6.1	14.25 ± 4.88	0.396
LEDD total	1120.91 ± 492.06	1224.11 ± 461.91	0.113

Data are in mean ± standard deviation, unless otherwise specified.

Abbreviations: DA, dopamine receptor agonist; LD, Levodopa; LEDD total, levodopa equivalent daily dose total; NMSS, Non‐Motor Symptoms Scale; UPDRS‐III, Unified Parkinson's Disease Rating Scale, Part III.

The overall efficacy of STN DBS in the whole cohort was supported by the significant mean reduction in UPDRS‐IV and LEDD total in both the intention‐to‐treat (−3.74, 95% confidence interval [CI] −4.82 to −2.65, *P* < 0.001; and −728.96, 95% CI −536.33 to −921.59, *P* < 0.001, respectively) and per‐protocol population (−3.28, 95% CI −4.55 to −2.01, *P* < 0.001; and −797.04, 95% CI −560.85 to −1033.24, *P* < 0.001).

Stimulation parameters did not differ between treatment groups in either the intention‐to‐treat or per‐protocol populations (all *P* > 0.5) (Supplementary Table S1).

### Intention‐to‐Treat Analysis

Randomization to either DA or LD did not significantly affect the primary outcome (*P* = 0.566) (Table 2). As for secondary outcomes, patients on DA had a greater increase in AESs and a trend toward significance for a greater increase in AESc compared to patients on LD (*P* = 0.019 and *P* = 0.060, respectively). In keeping with our study design, patients on DA had a greater reduction in LEDD LD and a greater increase in LEDD DA compared to patients on LD (*P* = 0.019 and *P* = 0.035, respectively) (Fig. 1B).

**TABLE 2:** Primary and secondary outcomes at baseline and 3 months after surgery (intention‐to‐treat population)

	Baseline		Three months		Mean change value comparison, DA–LD (95% CI)*	*P*
	DA (=18)	LD (=17)	DA (=18)	LD (=17)		
NMSS	59.83 ± 46.08	43.42 ± 25.31	52.3 ± 52.36	43.35 ± 23.71	4.88 (−11.78–21.53)	0.566
UPDRS‐III^a^	12.16 ± 6.1	14.25 ± 4.88	14.4 ± 5.68	18.42 ± 6.24	1.35 (−4.43–1.71)	0.386
UPDRS‐II	6 ± 4.43	5.5 ± 3.06	5.4 ± 3.94	10.14 ± 5.3	−1.36 (−4.07 to 1.34)	0.323
UPDRS‐IV	4.11 ± 2.31	6.25 ± 1.95	1.8 ± 1.31	3.64 ± 1.94	−0.71 (−2.03 to 0.61)	0.293
PDQ‐39	27.84 ± 17.02	23.04 ± 10.69	23.01 ± 14.63	25.23 ± 12.77	−2.28 (−8.4 to 3.85)	0.470
HAD‐A	5.33 ± 3.33	4.67 ± 2.6	4.9 ± 2.8	5.42 ± 3.41	0.05 (−1.52 to 1.62)	0.954
HAD‐D	4.33 ± 4.16	3.75 ± 2.17	5.1 ± 4.67	5 ± 3.55	0.08 (−1.67 to 1.82)	0.932
AESs	15.75 ± 14.36	13.25 ± 15.52	28.9 ± 19.9	13.21 ± 7.36	9.10 (1.52 to 16.67)	**0.019**
AESc	19 ± 16.9	11.83 ± 14.8	27.6 ± 19.33	16.64 ± 11.46	7.77 (−0.32 to 15.86)	0.060
QUIP	8.25 ± 6.78	14.25 ± 20.8	14.9 ± 16.14	11.5 ± 14.12	0.10 (−6.81 to 7.01)	0.977
PDSS	20.75 ± 11.97	20.25 ± 11.73	14.6 ± 8.42	18.14 ± 9.31	−3.37 (−8.22 to 1.47)	0.172
LEDD total	1326.33 ± 507.63	1483.23 ± 528.6	576.52 ± 380.63	900.64 ± 542.96	−229.79 (−506.32 to 46.72)	0.103
LEDD DA	205.41 ± 156.43	259.11 ± 129.83	231.17 ± 171.86	18.52 ± 36.56	79.1 (5.51 to 152.69)	**0.035**
LEDD LD	1120.91 ± 492.06	1224.11 ± 461.91	351.23 ± 375.64	885.05 ± 537.52	−307.51 (−565.18 to −49.84)	**0.019**

Data are expressed as mean ± standard deviation, unless otherwise specified.

^*^ Mean change value comparison shows the mean difference (95% CI) of DA compared to LD over the follow‐up (unpaired *t* test).

^a^ Med on before surgery and med on/stim on after surgery.

Abbreviations: AESc, Apathy Evaluation Scale caregiver‐administered; AESs, Apathy Evaluation Scale self‐administered; CI, confidence interval; DA, dopamine receptor agonist; HAD‐A, Hospital Anxiety Depression Scale—anxiety subscore; HAD‐D, Hospital Anxiety Depression Scale—depression subscore; LD, levodopa; LEDD total, levodopa equivalent daily dose total; LEDD DA, levodopa equivalent daily dose dopamine agonist; LEDD LD, levodopa equivalent daily dose levodopa; NMSS, Non‐Motor Symptoms Scale; PDQ‐39, 39‐Item Parkinson's Disease Questionnaire; PDSS, Parkinson's Disease Sleep Scale; QUIP, Questionnaire for Impulsive‐Compulsive Disorders in Parkinson's Disease; UPDRS‐II, Unified Parkinson's Disease Rating Scale, Part II; UPDRS‐III, Unified Parkinson's Disease Rating Scale, Part III; UPDRS‐IV, Unified Parkinson's Disease Rating Scale, Part IV.Bold‐typed values represent statistically significant findings.

No other significant differences according to treatment arm were detected for the remaining outcome variables (Table 2).

### Per‐Protocol Analysis

Randomization to DA monotherapy did not have a significant impact on NMSS (*P* = 0.710) (Table 3). As the baseline UPDRS‐III was significantly lower in DA than in LD (8 ± 3 vs. 14.5 ± 4.76, *P* = 0.004), it was added as a covariate in the subsequent analyses. No other differences were present at baseline (Table 3).

**TABLE 3:** Primary and secondary outcomes at baseline and 3 months after surgery (per‐protocol analysis)

	Baseline		Three months		Mean change value comparison, DA–LD (95% CI)*	*P*
	DA (=7)	LD (=14)	DA (=7)	LD (=14)		
NMSS	39.5 ± 25.09	48.5 ± 24.42	40.5 ± 40.4	44.18 ± 25.47	−3.79 (−23.76 to 16.18)	0.710
UPDRS‐II	5.83 ± 4.11	5.5 ± 3.3	4.83 ± 4.57	9.63 ± 5.88	−3.07 (−5.94 to −0.20)	**0.036**
UPDRS‐IV	5 ± 2.75	6.4 ± 2.01	1.33 ± 1.21	3.54 ± 2.06	−2.09 (−3.23 to −0.35)	**0.019**
PDQ‐39	16.23 ± 5.76	22.3 ± 12.93	11.73 ± 4.61	22.6 ± 11.99	−7.34 (−14.76 to 0.08)	0.053
HAD‐A	3.33 ± 3.14	5 ± 2.7	3.16 ± 1.83	4.36 ± 2.83	−1.10 (−3.21 to 0.99)	0.302
HAD‐D	2.17 ± 1.32	4.2 ± 2.09	2.33 ± 1.96	5 ± 3.97	−0.92 (−3.15 to −1.31)	0.419
AESs	14.67 ± 18.56	8.5 ± 5.16	31.5 ± 24.6	13.54 ± 8.2	11.54 (0.28 to 4.04)	**0.044**
AESc	22.33 ± 20.93	8.10 ± 4.33	32 ± 22.17	17.36 ± 12.67	16.43 (4.8 to 28.07)	**0.006**
QUIP	6.67 ± 6.28	15.6 ± 22.53	4.66 ± 4.08	12.63 ± 15.8	−5.77 (−16.99 to 5.44)	0.313
PDSS	17 ± 10.56	22.5 ± 11.59	12.66 ± 8.64	17.18 ± 9.15	−7.02 (−13.98 to −0.06)	**0.048**
LEDD total	1346.28 ± 677.68	1505.71 ± 452	297.42 ± 143.22	837.571 ± 504.55	−347.385 (729.71 to 34.94)	0.075

Data are expressed as mean ± standard deviation, unless otherwise specified.

^*^ Mean change value comparison shows the mean difference (95% CI) in DA compared to LD over the follow‐up (unpaired *t* test), including baseline UPDRS‐III as covariate.

Abbreviations: AESc, Apathy Evaluation Scale caregiver‐administered; AESs, Apathy Evaluation Scale self‐administered; CI, confidence interval; DA, dopamine receptor agonist; HAD‐A, Hospital Anxiety Depression Scale—anxiety subscore; HAD‐D, Hospital Anxiety Depression Scale—depression subscore; LD, levodopa; NMSS, Non‐Motor Symptoms Scale; PDQ‐39, 39‐Item Parkinson's Disease Questionnaire; PDSS, Parkinson's Disease Sleep Scale; QUIP, Questionnaire for Impulsive‐Compulsive Disorders in Parkinson's Disease; UPDRS‐II, Unified Parkinson's Disease Rating Scale, Part II; UPDRS‐III, Unified Parkinson's Disease Rating Scale, Part III; UPDRS‐IV, Unified Parkinson's Disease Rating Scale, Part IV; LEDD, levodopa equivalent daily dose.Bold‐typed values represent statistically significant findings.

As for secondary outcomes, patients on DA had a greater decrease in UPDRS‐II (*P* = 0.036), UPDRS‐IV (*P* = 0.019), and PDSS (*P* = 0.048) and a greater increase in AESs (*P* = 0.044) and AESc (*P* = 0.006) compared to patients on LD. Otherwise, no significant differences according to treatment arm were found for other variables (Table 3). There was a trend toward significance for greater reduction in LEDD total in DA (*P* = 0.075).

### Capacity to Maintain Monotherapy

**FIG. 2:**
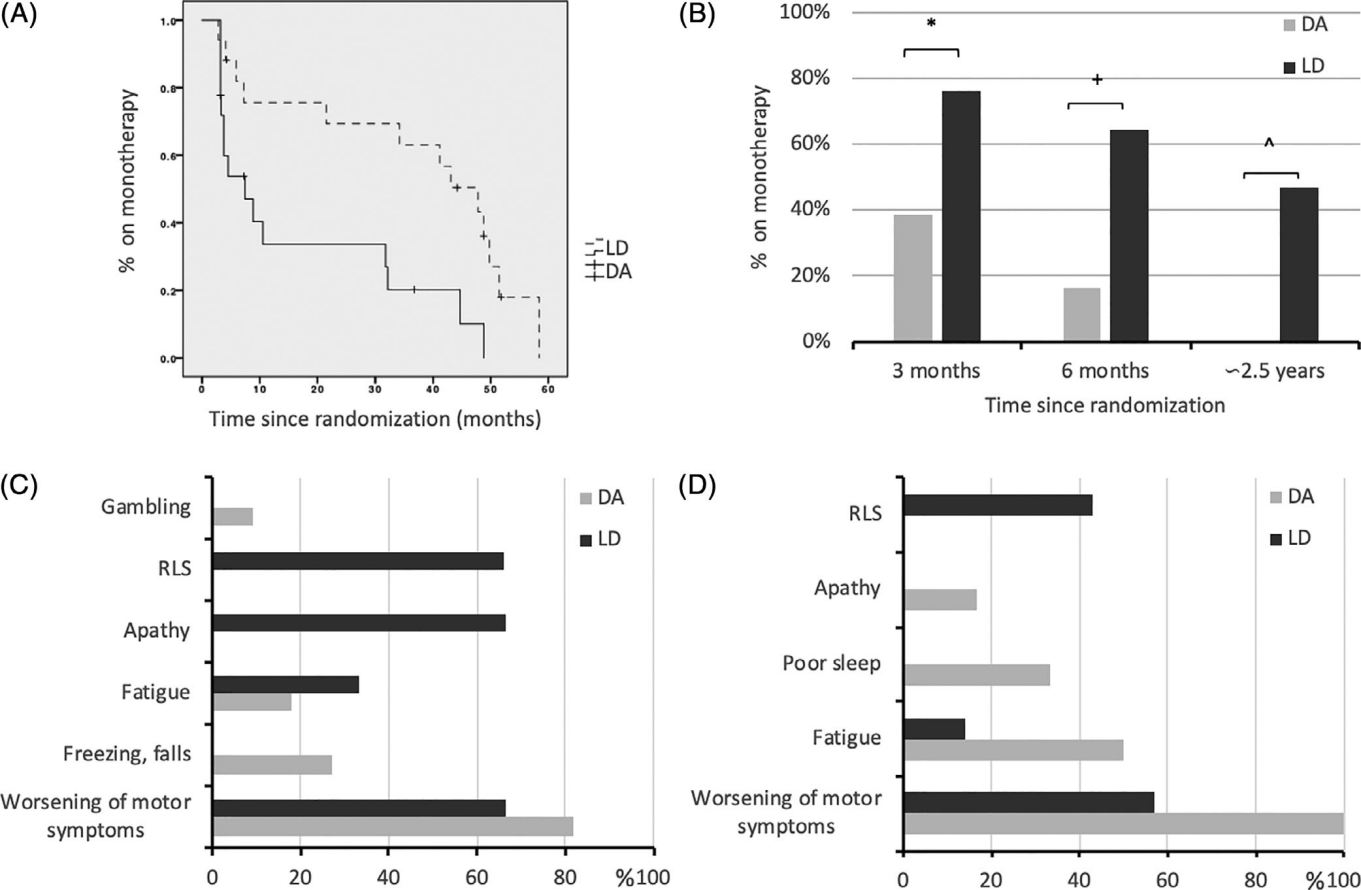
(**A**) Time to monotherapy failure according to the treatment arm. Dotted line: levodopa monotherapy; solid line: dopamine agonist monotherapy. (**B**) Capacity to maintain monotherapy at short‐, mid‐, and long‐term follow‐up. *, *P* = 0.022; +, *P* = 0.010; ^, *P* < 0.001. Reasons for monotherapy failure at (**C**) 3‐month and (**D**) 6‐month follow‐up in the intention‐to‐treat population. Each patient could report more than 1 reason. DA, dopamine receptor agonist; LD, levodopa; RLS, restless leg syndrome.

The median time to monotherapy failure for the overall population was 31.8 months (95% CI: 4.7–58.9), and it was significantly shorter for the DA arm compared to the LD arm (7.4 months, 95% CI: 1.2–13.5 vs. 47.8 months, 95% CI: 35.7–59.9, *P* = 0.005, Fig. 2A).

Fourteen of 35 patients (40%) failed to maintain monotherapy at 3‐month postoperative follow‐up. In detail, 3 of 17 (17.6%) patients assigned to LD monotherapy failed to wean off DA, and 11 of 18 (61.1%) patients assigned to DA monotherapy failed to wean off LD (*P* = 0.022) (Fig. 2B). Adverse events leading to 3‐month failure are detailed in Figure 2C. Multivariate logistic regression analysis showed that the only significant predictors of monotherapy failure 3 months after surgery were assigned to the DA arm (OR = 6.4, 95% CI 1.10–47.5, *P* = 0.047) and the distance between active DBS electrode contact and STN on the medial–lateral axis (OR = 2.67, 95% CI 0.66–5.65, *P* = 0.031), indicating an association between 3‐month monotherapy success and lateral STN stimulation (success: 1.37 ± 0.51 mm, failure: 0.86 ± SD 0.38 mm). Post hoc analyses performed separately for the DA and LD monotherapy treatment groups indicated that active DBS electrode contact was significantly more lateral in the DA patients who were able to maintain the monotherapy treatment (active contact–STN distance in 3‐month monotherapy success: 1.52 ± 0.74 mm, failure: 0.83 ± 0.38 mm, *P* = 0.02). This effect was not significant in the LD group (success: 1.29 ± 0.36 mm, failure: 0.95 ± 0.48 mm, *P* = 0.20). The detailed results from the univariate regression are available in Supplementary Table S2.

At 6‐month follow‐up, 17 of 35 patients (48.5%) failed to maintain monotherapy. In detail, 6 of 17 (35.2%) patients assigned to LD monotherapy failed to wean off DA, and 15 of 18 (83.3%) patients assigned to DA monotherapy failed to wean off LD (*P* = 0.010). Adverse events motivating 6‐month failure are shown in Figure 2D.

### Long‐Term Follow‐Up

After 31.4 ± 6.3 months postoperatively, the overall efficacy of STN DBS was confirmed by the significant reduction in LEDD total (−469.8, 95% CI −23.56 to −700.04, *P* < 0.001). Eight of 11 patients randomly assigned to LD were still receiving monotherapy at 6 months postoperatively. Three patients had to restart DA due to behavioral issues (apathy and depressed mood) and reported clinical improvement.

On the other hand, none of the 3 patients randomly assigned to DA and still on monotherapy at 6‐month follow‐up was receiving monotherapy. One patient had to add LD for freezing and falls with reported clinical improvement, whereas the remaining 2 patients, who had no history of behavioral problems before DBS, had to convert to LD monotherapy due to new onset ICDs.

In summary, 8 of 17 (47.1%) and 0 of 18 (0%) could maintain LD and DA monotherapy, respectively, based on initial randomization (*P* < 0.001). Regardless of the initial randomization, 12 of 35 patients (34.2%) were on LD monotherapy, and none were on DA monotherapy.

## Discussion

This clinical trial compares the efficacy of LD and DA monotherapy after STN DBS. Overall, our findings suggest that in short‐ and mid‐term follow‐up, monotherapy after surgery is safe and feasible in more than half of patients (60% at 3‐month and 51.5% at 6‐month follow‐up). However, over long‐term follow‐up, LD is the only feasible monotherapy in a minority of patients (34.2%), whereas DA monotherapy is not tolerated and is associated with the development of ICDs in 22.2% of patients.

The significant improvement in motor complications and LEDD confirmed the efficacy of STN DBS, irrespective of treatment arm assignment.^2^, ^3^ However, we failed to prove our initial hypothesis that patients on DA monotherapy would have greater improvement in NMS. The per‐protocol analysis of secondary outcomes demonstrated that DA monotherapy allowed greater improvement in activities of daily living, motor complications, and sleep quality compared to LD (Table 3). However, although these analyses included UPDRS‐III as a covariate, we acknowledge such results may simply reflect the better preoperative motor status of the per‐protocol DA population demonstrated by lower UPDRS‐III at baseline. This is not surprising with regard to the observation that the need for LD is considerably a surrogate marker of disease progression in de novo PD patients.^26^ Our data would therefore suggest that DA monotherapy is safe and feasible in a small proportion of patients over the short‐ and mid‐terms (about 40% at 3‐month follow‐up but less than 20% at 6‐month follow‐up) with better preoperative disease features and contact location within the dorsolateral STN as well as excellent DBS outcomes. Accordingly, the multivariate logistic regression analysis showed that a significant predictor of monotherapy failure 3 months after surgery was the assignment to the DA arm. As such, any patient assigned to DA had a sixfold greater risk to fail monotherapy compared with those assigned to LD. In addition, the success of maintaining DA monotherapy was significantly related to more lateral DBS electrode contact localization, confirming that the best clinical results are obtained when contacts are located in the dorsolateral STN border zone.^27^ Conversely, the pre‐DBS DA dose had no influence on maintaining monotherapy.

Adverse events leading to 3‐month DA monotherapy failure included worsening of motor symptoms (81.8%), freezing of gait and balance issues (27.2%), fatigue (18.1%), and gambling (9%). As expected, discontinuing LD induced a worsening of motor symptoms, freezing, and balance problems in a subgroup of patients who did not tolerate DA monotherapy. In addition, we expected the occurrence of gambling in 1 patient, although we demonstrated overall safety of DA therapy at 3‐month follow‐up, because QUIP scores did not differ between DA and LD monotherapy in both the intention‐to‐treat and per‐protocol analyses. This is probably due to the overall reduction in LEDD and the selection bias of not offering STN DBS in patients with active ICDs.^28^ Of note, on long‐term follow‐up, 4 patients assigned to DA (22.2%) developed significant ICDs requiring the discontinuation of DA. More surprising is the occurrence of fatigue in this cohort, thus meaning that LD can also improve such NMS.^29^

Surprisingly, in both the intention‐to‐treat and per‐protocol analyses, there was a greater increase in apathy severity in the DA group as evaluated by both patients and caregivers. We speculate that such findings may reflect the effect of the overall dopaminergic load (ie, LEDD total) on such specific mood symptoms as apathy.^30^ Thus, there was a trend toward significance for lower LEDD total for DA compared to LD in both intention‐to‐treat and per‐protocol analyses. An effect of STN DBS in inducing loss of motivation independently from the dopaminergic neurodegenerative process or the reduction in dopamine replacement therapy has to be considered.^31^ Nevertheless, our analysis demonstrated that patients with dorsolateral STN electrode placement could maintain DA monotherapy, thus resulting in an overall low LEDD.

In the per‐protocol analysis, DA monotherapy was associated with significantly lower UPDRS‐IV scores, probably with regard to a lower amount of dyskinesias induced by DA compared to LD monotherapy. The DA group showed significantly lower PDSS scores, reflecting greater sleep quality. Better motor status and lower risk of restless leg syndrome (RLS) in DA may contribute to this finding.^1^, ^2^, ^3^, ^4^

On the contrary, LD monotherapy can be maintained by a greater proportion of patients in the short‐ and mid‐terms (in 82.4% and 64.8% of patients, respectively), but only 34.2% of them can maintain it in the long term, in keeping with a previous retrospective study.^5^ Intriguingly, we failed to find the same relationship with contact location for patients on LD monotherapy, suggesting that LD alone can provide a motor benefit, even when electrodes are not optimally placed.

The reasons for the 3‐month LD monotherapy failure include apathy, worsening of motor symptoms, RLS (66.6% each), and fatigue (33.3%). As expected, discontinuing DA induced apathy and RLS in a subgroup of patients who did not tolerate LD monotherapy. Surprisingly, half of patients who failed to wean off DA complained of worsening of motor symptoms at 6‐month follow‐up, thus suggesting the role of the total dopaminergic load in influencing the postoperative outcome in terms of both motor symptoms and NMS.

### Strengths and Limitations

This prospective, randomized, single‐blind clinical trial explored the efficacy and safety of LD versus DA monotherapy after STN DBS in PD. A comprehensive motor and non‐motor evaluation was applied to a cohort enrolled in a single center, ensuring homogeneity of population and evaluation but limiting external validity of the findings. The enrolled cohort was followed up to 2.5 years after surgery to describe the efficacy and safety of antiparkinsonian monotherapy. We were also able to show a relationship between electrode location and ability to maintain monotherapy.

The limited number of participants in the per‐protocol analysis demonstrates that it is difficult to adhere to the proposed strategy (ie, monotherapy after STN DBS). Nevertheless, the intention‐to‐treat analysis compared 2 groups with significant differences in terms of LEDD DA and LEDD LD. Overall, these findings show that we were able to compare two different therapeutic strategies, thus gathering evidence from a group of patients who fulfilled the requirements of our sample size calculation. We acknowledge some heterogeneity in the enrolled cohort, as 3 of 35 patients underwent a unilateral procedure. However, the repeat analysis excluding those subjects did not change the study results (Supplementary Tables [Link], [Link]).

## Conclusion

In conclusion, this trial showed that DA monotherapy is not more effective in improving NMS compared to LD monotherapy after STN DBS. More important, our findings suggest that in short‐ and mid‐term follow‐up, monotherapy after surgery is safe and feasible in more than half of patients, particularly for patients on LD. The ability to maintain DA monotherapy in the short term is related to lateral STN contact location. Furthermore, LD monotherapy is feasible only in a minority of patients (34.2%) in the long term, whereas DA monotherapy is not tolerated and is associated with the development of ICDs in a significant proportion of patients.

Although more studies are certainly warranted, the result of our trial is an initial step toward evidence‐based management of medications after STN DBS. We can conclude that physicians should aim at reducing DA while monitoring the occurrence of NMS such as apathy and RLS. Nevertheless, although simplification of therapy is always warranted, for most patients (eg, those on high doses of DA before surgery), a combination of LD and DA is still the best way to guarantee a successful outcome after DBS.

## Author Roles

(1) Research project: A. Conception, B. Organization, C. Execution; (2) Statistical analysis: A. Design, B. Execution, C. Review and critique; (3) Manuscript preparation: A. Writing of the first draft, B. Review and critique.

M.P.: 1B, 1C, 2A, 2B, 3A

O.P., Y.‐Y.P., C.C.M., R.P.M., S.K.K., M.H., A.M.L.: 1C, 2C, 3B

S.B.B.: 1C, 2B, 2C, 3B

A.F.:1A, 1B, 2C, 3B

## Authors' Contributions

1. Research project: A. Conception, B. Organization, C. Execution;

2. Statistical Analysis: A. Design, B. Execution, C. Review and Critique;

3. Manuscript Preparation: A. Writing of the first draft, B. Review and Critique;

MP: 1B, 1C, 2A, 2B, 3A

OJ, YYP, CCM, RPM, SKK, MH, AML: 1C, 2C, 3B

SBB: 1C, 2B, 2C, 3B

AF:1A, 1B, 2C, 3B

## Full financial disclosures for the previous 12 months

C.C.M. receives consultant fees from Boston Scientific Neuromodulation and royalties from Hologram Consultants, Neuros Medical, and Qr8 Health and is a shareholder in the following companies: Hologram Consultants, Surgical Information Sciences, CereGate, Autonomic Technologies, Cardionomic, and Enspire DBS; the other authors declare no conflict of interest. S.K.K. is a consultant for Medtronic (which manufactures the hardware implanted in patients included in the present study); A.M.L. received honoraria from Abbott, Boston Scientific, and Medtronic; A.F. received honoraria from Abbott, Boston Scientific, Brainlab, Medtronic, and UCB pharma.

## Supporting information


**Table S1.** Three‐month postoperative stimulation parameters for the intention‐to‐treat and per‐protocol population according to randomization.

**Table S2.** Predictors of 3‐month postoperative monotherapy failure: univariate regression (all subjects).

**Table S3.** Changes in primary and secondary outcomes from baseline to 3 months (intention‐to‐treat population excluding 3 patients who underwent unilateral procedures).

**Table S4.** Predictors of 3‐month postoperative monotherapy failure: univariate regression (excluding 3 patients who underwent unilateral procedures).

**Table S5.** Predictors of 3‐month postoperative monotherapy failure: multivariate regression (excluding 3 patients who underwent unilateral procedures).
