# Supplementary material for: Levodopa Versus Dopamine Agonist after Subthalamic Stimulation in Parkinson's Disease
Source: Mov Disord. 2020 Nov 9;36(3):672–80. doi: 10.1002/mds.28382 (PMC8048876; doi:10.1002/mds.28382)
Supplement: Supplementary file 1 — Table S1. Three‐month postoperative stimulation parameters for the intention‐to‐treat and per‐protocol population according to randomization. [file MDS-36-672-s001.docx]

**Suppl. Table 1.** Three-month post-operative stimulation parameters for the intention-to-treat and per-protocol population according to randomization.

|  | **Intention-to-treat** | | | **Per-protocol** | | |
| --- | --- | --- | --- | --- | --- | --- |
|  | **LD (N = 17)** | **DA (N = 18)** | **p** | **LD (N = 14)** | **DA (N = 7)** | **p** |
| **Right amplitude, V** | 2.94 ± 0.76 | 2.85 ± 1 | 0.842 | 2.84 ± 0.82 | 2.71 ± 1.19 | 0.786 |
| **Right pulse width, μsec** | 62 ± 7.74 | 60 ± 0.0 | 0.310 | 62.5 ± 8.66 | 60 ± 0.0 | 0.461 |
| **Right frequency, Hz** | 130.66 ± 25.13 | 146.56 ± 30.48 | 0.125 | 130.83 ± 28.35 | 149.28 ± 33.7 | 0.218 |
| **Left amplitude, V** | 3.1 ± 0.69 | 2.62 ± 1.06 | 0.150 | 3.08 ± 0.76 | 2.48 ± 1.34 | 0.239 |
| **Left pulse width, μsec** | 64 ± 10.55 | 60 ± 0.0 | 0.153 | 65 ± 11.67 | 60 ± 0.0 | 0.317 |
| **Left frequency, Hz** | 130.66 ± 25.13 | 142.33 ± 26.24 | 0.224 | 130.83 ± 28.35 | 139.16 ± 22.45 | 0.541 |
| **Monopolar configuration, n (%)** | 26 (81.5) | 34 (97.1) | 0.084 | 23 (95.8) | 12 (92.3) | 0.757 |

Data are in mean ± standard deviation, unless otherwise specified. Percentage of monopolar configuration computed on a total of 32 electrodes for LD and 35 electrodes for DA for intention-to-treat population and on a total of 24 electrodes for LD and 13 electrodes for DA for per-protocol population. Abbreviations: DA: dopamine receptor agonist; LD: Levodopa.
