# Supplementary material for: Levodopa Versus Dopamine Agonist after Subthalamic Stimulation in Parkinson's Disease
Source: Mov Disord. 2020 Nov 9;36(3):672–80. doi: 10.1002/mds.28382 (PMC8048876; doi:10.1002/mds.28382)
Supplement: Supplementary file 2 — Table S2. Predictors of 3‐month postoperative monotherapy failure: univariate regression (all subjects). [file MDS-36-672-s005.docx]

**Suppl. Table 2.** Predictors of 3-month post-operative monotherapy failure: univariate regression (all subjects).

| **Independent variable** | **OR (95%CI)** | **p** |
| --- | --- | --- |
| Age | 0.96 (0.89 to 1.04) | 0.377 |
| Disease duration | 0.93 (0.78 to 1.11) | 0.449 |
| UPDRS-III | 1.11 (0.97 to 1.26) | 0.117 |
| LEDD | 0.99 (0.99 to 1.01) | 0.379 |
| LEDD LD | 1 (0.99 to 1) | 0.573 |
| LEDD DA | 0.99 (0.99 to 1) | 0.198 |
| Weight | 1.12 (0.98 to 1.07) | 0.219 |
| Randomization | 5.1 (1.17 to 11.54 | 0.029 |
| Active DBS electrode contact-STN distance | 4.28 (1.42 to 8.60) | 0.016 |

Abbreviations: CI: confidence interval; DA: dopamine agonist; DBS: deep brain stimulation; LD: levodopa; LEDD: levodopa equivalent daily dose; OR: odds ratio.
