# Supplementary material for: Levodopa Versus Dopamine Agonist after Subthalamic Stimulation in Parkinson's Disease
Source: Mov Disord. 2020 Nov 9;36(3):672–80. doi: 10.1002/mds.28382 (PMC8048876; doi:10.1002/mds.28382)
Supplement: Supplementary file 3 — Table S3. Changes in primary and secondary outcomes from baseline to 3 months (intention‐to‐treat population excluding 3 patients who underwent unilateral procedures). [file MDS-36-672-s004.docx]

**Suppl Table 3.** Changes in primary and secondary outcomes from baseline to three months (Intention-to-treat population excluding three patients who underwent unilateral procedures).

|  | **Pairwise comparison**  **DA-LD (95%CI)** | **p** |
| --- | --- | --- |
|  |  |  |
| NMSS | 3.44 (-14.20 to 21.07) | 0.703 |
| UPDRS-III | 1.35 (-4.41 to 1.95) | 0.450 |
| UPDRS-II | -0.92 (-3.81 to 1.96) | 0.530 |
| UPDRS-IV | -0.52 (-1.93 to 0.89) | 0.470 |
| PDQ-39 | -15.85 (-68.9 to 37.2) | 0.558 |
| HAD-A | -0.13 (-1.81 to 1.55) | 0.879 |
| HAD-D | 0.01 (-1.85 to 1.85) | 0.999 |
| AESs | 9.15 (1.01 to 17.28) | **0.027** |
| AESc | 7.28 (-1.06 to 15.62) | 0.087 |
| QUIP | -0.18 (-4.64 to 4.28) | 0.937 |
| PDSS | -3.19 (-8.28 to 1.9) | 0.220 |
| LEDD Total | -156.62 (-437.68 to 124.43) | 0.275 |
| LEDD DA | 79.7 (0.85 to 158.57) | **0.048** |
| LEDD LD | -234.97 (-497.01 to -27.07) | **0.049** |

Pairwise comparison shows the mean difference (95%CI) of DA compared to LD. Abbreviations: AESc: Apathy Evaluation Scale caregiver-administered; AESs: Apathy Evaluation Scale self-administered; CI: confidence interval; DA: dopamine receptor agonist; HAD-A: the Hospital Anxiety Depression Scale – anxiety subscore; HAD-A: the Hospital Anxiety Depression Scale – depression subscore; LD: levodopa; LEDD total: levodopa equivalent daily dose total; LEDD DA: levodopa equivalent daily dose dopamine receptor agonists dose; LEDD LD: levodopa equivalent daily dose levodopa dose; NMSS: Non-Motor Symptoms ScalePDQ39: 39-item Parkinson’s disease questionnaire; PDSS: Parkinson's disease Sleep Scale; QUIP: Questionnaire for Impulsive-Compulsive Disorders in Parkinson’s Disease; UPDRS-II: Unified Parkinson’s disease rating scale part II; UPDRS-III: Unified Parkinson’s disease rating scale part III; UPDRS-IV: Unified Parkinson’s disease rating scale part IV.
