# Supplementary material for: Levodopa Versus Dopamine Agonist after Subthalamic Stimulation in Parkinson's Disease
Source: Mov Disord. 2020 Nov 9;36(3):672–80. doi: 10.1002/mds.28382 (PMC8048876; doi:10.1002/mds.28382)
Supplement: Supplementary file 4 — Table S4. Predictors of 3‐month postoperative monotherapy failure: univariate regression (excluding 3 patients who underwent unilateral procedures). [file MDS-36-672-s002.docx]

**Suppl Table 4.** Predictors of 3-month post-operative monotherapy failure: univariate regression (excluding three patients who underwent unilateral procedures).

| **Independent variable** | **OR (95%CI)** | **p** |
| --- | --- | --- |
| Age | 0.965(0.87 to 1.04) | 0.287 |
| Disease duration | 0.9 (0.74 to 1.09) | 0.293 |
| UPDRS-III | 1.08 (0.95 to 1.24) | 0.210 |
| LEDD | 0.99 (0.99 to 1.01) | 0.324 |
| LEDD LD | 1 (0.99 to 1) | 0.539 |
| LEDD DA | 0.99 (0.99 to 1) | 0.164 |
| Weight | 1.03 (0.98 to 1.08) | 0.183 |
| Randomization | 5.04 (1.10 to 22.96) | **0.037** |
| Active DBS electrode contact-STN distance | 3.85 (1.08 to 8.21) | **0.029** |

Abbreviations: CI: confidence interval; DA: dopamine agonist; DBS: deep brain stimulation; LD: levodopa; LEDD: levodopa equivalent daily dose; OR: odds ratio.
