# Supplementary material for: Levodopa Versus Dopamine Agonist after Subthalamic Stimulation in Parkinson's Disease
Source: Mov Disord. 2020 Nov 9;36(3):672–80. doi: 10.1002/mds.28382 (PMC8048876; doi:10.1002/mds.28382)
Supplement: Supplementary file 5 — Table S5. Predictors of 3‐month postoperative monotherapy failure: multivariate regression (excluding 3 patients who underwent unilateral procedures). [file MDS-36-672-s003.docx]

**Suppl. Table 5.** Predictors of 3-month post-operative monotherapy failure: multivariate regression (excluding three patients who underwent unilateral procedures).

| **Independent variable** | **OR (95%CI)** | **p** |
| --- | --- | --- |
| Randomization | 7.69 (1.23 to 66.52) | **0.039** |
| Active DBS electrode contact-STN distance | 2.58 (0.61 to 5.54) | **0.034** |

Abbreviations: CI: confidence interval; DBS: deep brain stimulation; OR: odds ratio.
